# A comprehensive analysis of the circRNA–miRNA–mRNA network in osteocyte-like cell associated with *Mycobacterium leprae* infection

**DOI:** 10.1371/journal.pntd.0010379

**Published:** 2022-05-02

**Authors:** Zheng-Rong Gao, Qiong Liu, Jie Zhao, Ya-Qiong Zhao, Li Tan, Shao-Hui Zhang, Ying-Hui Zhou, Yun Chen, Yue Guo, Yun-Zhi Feng

**Affiliations:** 1 Department of Stomatology, The Second Xiangya Hospital, Central South University, Changsha, Hunan, China; 2 National Clinical Research Center for Metabolic Diseases, Hunan Provincial Key Laboratory of Metabolic Bone Diseases, and Department of Metabolism and Endocrinology, The Second Xiangya Hospital of Central South University, Changsha, Hunan, China; NHDP: National Hansen’s Disease Program, UNITED STATES

## Abstract

**Background:**

Bone formation and loss are the characteristic clinical manifestations of leprosy, but the mechanisms underlying the bone remodeling with *Mycobacterium leprae* (*M*. *leprae*) infection are unclear.

**Methodology/Principal findings:**

Osteocytes may have a role through regulating the differentiation of osteogenic lineages. To investigate osteocyte-related mechanisms in leprosy, we treated osteocyte-like cell with N-glycosylated muramyl dipeptide (N.g MDP). RNA-seq analysis showed 724 differentially expressed messenger RNAs (mRNAs) and 724 differentially expressed circular RNA (circRNAs). Of these, we filtered through eight osteogenic-related differentially expressed genes, according to the characteristic of competing endogenous RNA, PubMed databases, and bioinformatic analysis, including TargetScan, Gene Ontology, and Kyoto Encyclopedia of Genes and Genomes. Based on these results, we built a circRNA–microRNA (miRNA)–mRNA triple network. Quantitative reverse-transcription polymerase chain reaction and western blots analyses confirmed decreased *Clock* expression in osteocyte-like cell, while increased in bone mesenchymal stem cells (BMSCs), implicating a crucial factor in osteogenic differentiation. Immunohistochemistry showed obviously increased expression of CLOCK protein in BMSCs and osteoblasts in N.g MDP–treated mice, but decreased expression in osteocytes.

**Conclusions/Significance:**

This analytical method provided a basis for the relationship between N.g MDP and remodeling in osteocytes, and the circRNA–miRNA–mRNA triple network may offer a new target for leprosy therapeutics.

## Author summary

Although the number of leprosy patients is gradually decreasing after the use of rifampin, those who have been cured still have bone damage. The *Mycobacterium leprae* is difficult to culture *in vivo*, and the potential biosafe problem should be considered. Thus, the minimal essential structure in *Mycobacterium*, N-glycosylated muramyl dipeptide, has been used to reveal the mechanism in leprosy. According to the analysis results, various differentially expressed genes have been filtered, and *circadian Clock* gene might be the key factor. We verified that *Clock* gene was upregulated in the process of osteogenesis. Osteocytes would maintain the balance between osteoblasts and osteoclasts in bone homeostasis. We noticed that the expression of *Clock* gene was decreased after treated with N-glycosylated muramyl dipeptide. This might be the reason why bone damage is still maintained in the cured patient, and regulating the expression of target at posttranscriptional level by microRNAs and circular RNAs can be the promising therapeutic method in leprosy bone damage.

## Introduction

Leprosy, caused by the bacillus *Mycobacterium leprae* (*M*. *leprae*), is a chronic infectious disease, with more than 200,000 cases occurring annually worldwide [1]. Previous studies have shown that these patients are at risk for being misdiagnosed with other diseases, such as non-specified connective tissue disease and nodular vasculitis, delaying appropriate treatment [2]. The clinical manifestations of leprosy include lesions of the skin and peripheral nerves [3] and deformity and physical disability [4]. Poor oral hygiene also has been noted [5,6]. The mechanism underlying these clinical manifestations remain to be elaborated, however.

Leprosy leads to bone changes, but what causes these changes is controversial. Some researchers have suggested bone destruction as a likely explanation, and indeed, bone damage is observed in 90% of patients [7]. Furthermore, 40% of patients show absorption of terminal phalanges on radiology [8], resulting from the expression of a phosphate-regulating gene with homologies to endopeptidase on the X chromosome (PHEX) that facilitates suppression by *M*. *leprae* in osteoblasts [9]. Other authors, however, have reported finding bone formation in patients with leprosy [10]. Toll-like receptor (TLR)1 and TLR4 polymorphisms are associated with protection against leprosy [11,12], and mineralization-related protein is elevated in the condition [13]. Bone islands and osteosclerosis also have been identified by radiology in these patients [14,15]. Thus, the pathways leading to bone alteration in leprosy remain unclear.

Culturing *M*. *leprae in vitro* could crucially contribute to understanding of the bone remodeling mechanism, but this species cannot be cultured in artificial medium [16]. *M*. *tuberculosis* and *M*. *leprae* show phylogenetic proximity [17], and their bacterial products have been used in experimental analyses. Of these, muramyl dipeptide (MDP) is the minimal essential structure for the immunological effects of the cell wall [16] and can occur as *N*-acetyl MDP and N-glycosylated MDP (N.g MDP). N.g MDP is known to trigger an exceptionally strong immunogenic response [18].

Osteogenic lineages are key in modulating bone formation, and osteocytes especially play a central role in bone homeostasis [19]. Osteocytes produce and secrete molecules such as Dickkopf-1, fibroblast growth factor 23, dentine matrix protein-1 (DMP-1), PHEX, and matrix extracellular phosphoglycoprotein to mediate osteoblast differentiation, viability, and function [20–22]. In high-glucose conditions, osteocyte-derived exosomes carrying miR-124-3p can regulate Gal-3 expression of osteoblasts [23], and overexpression of Col1a2 in the osteocytes of insulin-receptor substrate-1–deficient mice (Irs-1^-/-^) can promote bone formation [24].

A large class of non-coding RNAs (ncRNAs) has been discovered through large-scale genome and transcriptome studies in recent years [25], including microRNAs (miRNAs) and circular RNAs (circRNAs). The miRNAs consist of ~22 nucleotides and have a crucial role in gene silencing [26]. Jorge et al. reported that miR-101, miR-196b, miR-27b, and miR-29c serve as biomarkers in the diagnosis of a subtype of leprosy [27], and Liu et al. found that through miRNA-21 expression, the host cell can ward off infection by *M*. *leprae*, preventing bacillus growth, generating natural barriers, and regulating the antibacterial pathway [28]. In addition, miR-342 mediates regulation of *Col1a2* expression in bone formation [24]. The circRNAs may have potentially important roles in gene regulation, as well [29]. These ncRNAs are produced through a downstream splice-donor site covalently linked to an upstream splice-acceptor site, in a process called backsplicing [30]. CircRNAs contain many miRNAs response elements and competing binding sites, thereby reducing miRNAs and mRNAs interaction and regulating the expression of target genes at the posttranscriptional level. Thus, it is more likely to have competing endogenous (ce)RNA function and act as miRNA sponges to restrain the expression of miRNA and regulate RNA transcription [30–32]. The circRNA Hsa_circ_0074834 has been associated with repair of bone defects and promotion of bone mesenchymal stem cells (BMSCs) osteogenic differentiation by acting as a ceRNA for miR-942-5p [33]. However, the involvement of circRNA in leprosy is unclear.

For a better understanding of gene expression in leprosy, the potential circRNA–miRNA–mRNA triple network needs to be investigated because it represents a possible therapeutic target. In this study, we constructed this triple network, using murine osteocyte-like MLO-Y4 cells treated with N.g MDP to elucidate the activity of this pathway in leprosy.

## Materials and methods

### Ethics statement

The study was approved by the Animal Ethics Committee of the Central South University (Approval No. 2021391) and conformed with the ARRIVE guidelines.

### Cell culture and treatment

MLO-Y4 cells, a murine osteocyte-like cell line, MC3T3-E1, a murine preosteoblast cell, were purchased from the Cell Bank of the Chinese Academy of Sciences (Shanghai, China). Cells were maintained at a density of 2.5 × 10^6^/well in 6-well plates kept in a humidified incubator at 5% CO_2_ at 37°C. Culture medium consisted of α-modified minimum essential medium (Gibco, Thermo Fisher Scientific, Inc., Waltham, MA, USA), supplemented with 1% Glutamax, 0.5% mycoplasma antibiotics, 10% fetal bovine serum (Gibco), and 1% penicillin and streptomycin. For the treated group, 1 μg/ml N-g MDP (Catalogue tlrl-gmdp, Invivogen) was applied for 36 h. Both BMSCs preparation and osteogenic induction have been reported before [24]. Briefly, BMSCs were cultured in osteogenic medium, which involved 10 mM β-glycerophosphate, 10^−8^ M dexamethasone and 50 μg/ml l-2-ascorbic acid.

### Animals

All protocols used in our animal experiments were approved by the Animal Ethics Committee of the Central South University and conformed with the ARRIVE guidelines. The C57BL/6 female mice were supplied by the Experimental Animal Center of Xiang-Ya Second Hospital. After a week of adjustable feeding, mice in the exposure group were infused with 2 μg N.g MDP dissolved in 100 μl of saline solution once a day for 10 days, and the control group was infused with 10 μl saline only. Both groups were euthanized with an overdose of anesthesia. We have previously published the details of femur removal [24]. Investigators were blinded to the group allocation during the experiment and when assessing the outcome.

### mRNA/circRNA sequencing

Total RNA was extracted from the samples in Trizol reagent according to the manufacturer’s instructions (Invitrogen). The concentration and purity of each RNA sample were determined using the dsDNA HS Assay kit for Qubit (12640ES76, Yeasen). The quality of the library was determined using an Agilent High Sensitivity DNA Kit (5067–4626, Agilent), and integrity and size were quantified with this kit on an Agilent 2100 Bioanalyser (Agilent Technologies, Santa Clara, CA). For the mRNA library, mRNA was purified via two rounds of hybridization to Dynal Oligo beads (N411-03, Vazyme). After depleting the samples of ribosomal RNAs, we applied fragmentation buffer (AM8740; Invitrogen) and synthesized cDNA from the fragments using random primers. Following end repair second-strand digestion and adaptor ligation, the purified fragments were PCR amplified. For the circRNA library, total RNA was subjected to ribosomal RNA depletion using the QIAseq FastSelect RNA Removal Kit (333180, QIAGEN). Remaining RNA samples were treated with RNase R (RNR07250; Epicenter) to remove linear RNAs. After preparation of cDNA, the remaining procedures were similar to those for the mRNA library. The library preparation was performed using the VAHTS mRNA-seq V3 Library Prep Kit for Illumina (NR611, Vazyme).

### Bioinformatics analysis

TargetScan (http://www.targetscan.org/) software packages were used to predict the potential miRNA target of the mRNAs. The biological processes involving Gene Ontology (GO) and Kyoto Encyclopedia of Genes and Genomes (KEGG) (adjusted P value < 0.05; gene count ≥2) were obtained from the database for Annotation, Visualization, and Integrated Discovery (known as DAVID) (https://david.ncifcrf.gov/) (gene count ≥2). The differentially expressed mRNAs and proteins were visualized using the Search Tool for the Retrieval of Interacting Genes (STRING) software (https://string-db.org/). The circRNA–miRNA–mRNA interaction network was built by merging the circRNA database (provided by Guangzhou Forevergen Biosciences), differentially expressed genes, and the predicted potential miRNA. The raw data has been submitted on the GEO database (PRJNA798506).

### qRT-PCR

After extraction, RNA (1 μg) was reverse-transcribed (PrimeScript RT kit; TaKaRa, Otsu, Japan) and qPCR performed (SYBR Premix Ex Taq II kit; TaKaRa) using the following primer sequences: Rcan2 forward, 5’-ACCTATGATGAATGTGTGACGT-3’, reverse, 5’ -TTAGCTTCTTCCCTCTGAACTG-3’; Cacnb4 forward, 5’-GTTTTACAGCGGCTGATTAAGT-3’, reverse, 5’ -TAACATCAAACATTTCAGGCGG-3’; Plag1 forward, 5’- CTTTCAGTGGGAAGCCTTGGGATG-3’, reverse, 5’ -GAACGCTGCCGACAGTGAGTG-3’; Lpar6 forward, 5’- ATCGTTTGCATTGCTGTGTGGTTC-3’, reverse, 5’ -GCAGGCTTCTGAGGTATTGTTCCC-3’; Npnt forward, 5’-GGTGATGGAGGACATGCGAATAGG-3’, reverse, 5’ -GTAGGCTGTGGTGTTGGGTTTGG-3’; Lin28b forward, 5’-GGAGACGGCAGGATTTACTGATGG-3’, reverse, 5’ -AATGGCACTTCTTTGGCTGAGGAG-3’; Clock forward, 5’- TTCCTGACCAAAGGCCAGCA-3’, reverse, 5’- CTCGCCGTCTTTCAGCCCTA-3’; Rora forward, 5’- TGGTGGAGTTTGCCAAACGC-3’, reverse, 5’- TGAGAGTCAAAGGCACGGCA-3’; and β-actin forward, 5’-CAACGAGCGGTTCCGATG-3’, reverse, 5’-GCCACAGGATTCCATACCCA-3’ (all Shanghai Generay Biotech Co., Ltd., Shanghai, China). Cycling conditions for PCR (LightCycler Real-time PCR System, Roche) were as follows: 30 s at 95°C for polymerase activation; 40 cycles of 5 s each at 95°C and of 20 s each at 60°C. Primer specificity was confirmed using melting curves, and the 2-ΔΔCt method was used to analyze the qRT-PCR results.

### Western blots

Western blots were performed as previously described [23]. Briefly, total protein extracts were prepared in RIPA buffer, which contained inhibitors of proteases and phosphatases. After electrophoresis, the SDS-PAGE–separated proteins were transferred to a polyvinylidene fluoride membrane (EMD–Millipore, Billerica, MA, USA). The membranes were blocked with QuickBlock (P0252, beyotime) for 20 min and incubated overnight at 4°C in 3% bovine serum albumin (9048-46-8, Sigma-Aldrich) in TBST supplemented with primary antibodies against CLOCK (AF0323, Affinity Biosciences), RUNX2 (ab23981, Abcam), Osteocalcin (PA5-86886, Invitrogen), and β-actin (AM1021B, Abcepta). Immunoreactive bands were detected using an antirabbit peroxidase–conjugated secondary antibody (1:5000; Bioss) and visualized with enhanced chemiluminescence (GE Healthcare, Little Chalfont, UK).

### Immunohistochemistry

Immunohistochemistry was performed as previously described [23]. Briefly, right femurs were fixed in 4% paraformaldehyde for 24–48 h, decalcified in 10% EDTA for 21 d, and embedded in paraffin. Decalcified right femur sections 5 μm thick were deparaffinized in turpentine, 3% H_2_O_2_ was used to suppress endogenous peroxidase activity, and the sections were treated with 0.1% trypsin for antigen retrieval. After incubation with a CLOCK polyclonal antibody (AF0323; 1:100, Affinity Biosciences) overnight, sections were incubated with secondary antibodies. Antibodies were detected by staining with a horseradish peroxidase–conjugated rabbit anti-mouse IgG and diaminobenzidine (GTVision III Detection System/Mo&Rb Kit; Gene Tech, Shanghai, China). Specimens were counterstained with hematoxylin.

### Statistical analysis

All experiments were carried out in triplicate. Data were analyzed using IBM SPSS Statistics 26. Statistical significance was considered at P < 0.05 using the Student’s t-test. The results are presented as mean ± standard deviation.

## Results

### Differentially expressed mRNAs and circRNAs in N.g MDP–treated osteocyte-like cell

**Fig 1:**
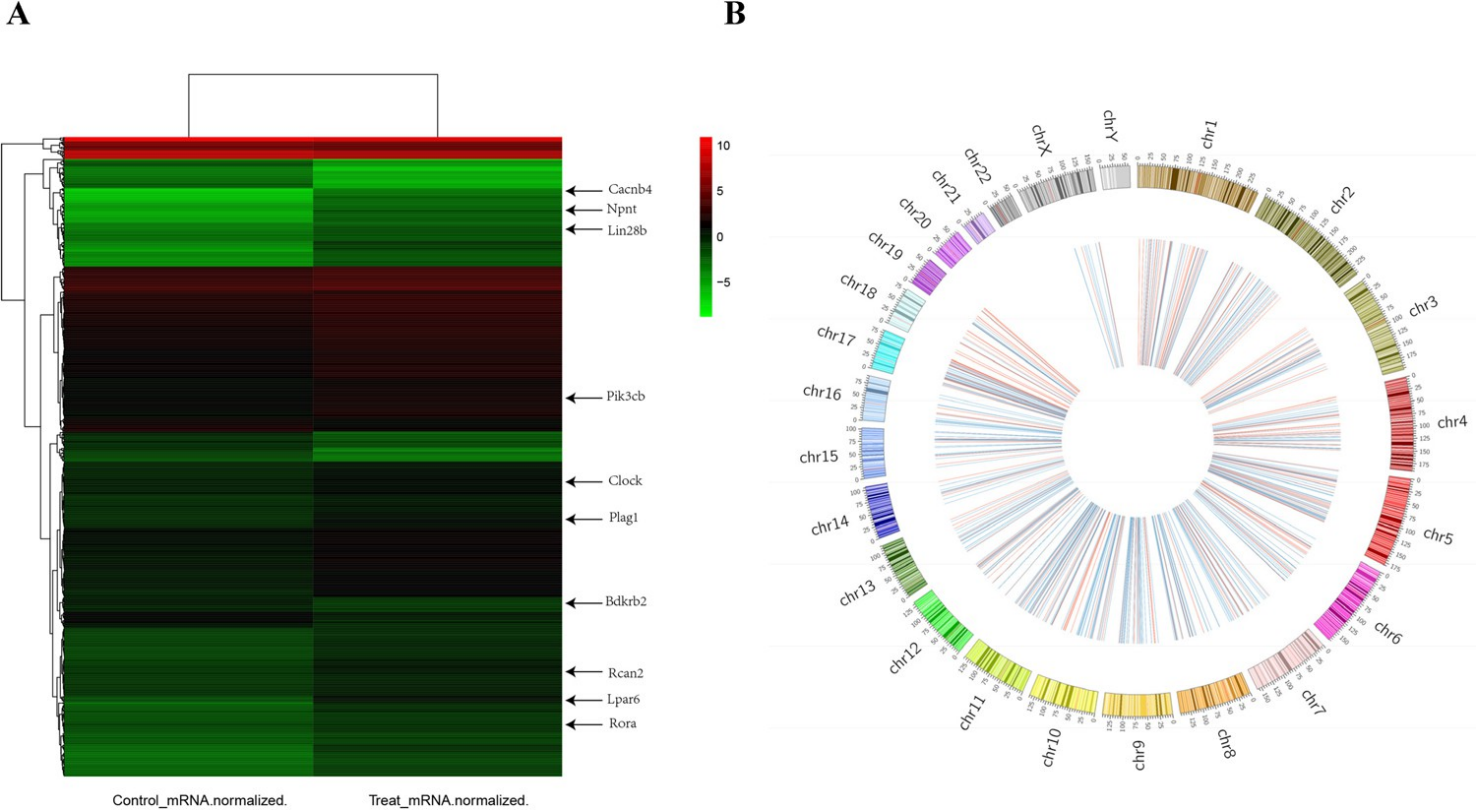
Identification of differentially expressed mRNAs and circRNAs. Heatmap of expression profiles of different mRNAs (A) and circRNAs (B), respectively (fold-change >1.5 and P < 0.05). Red and green/blue denote high and low relative expression, respectively.

Osteocyte-like cell was observed under a light microscope, and western blots showed DMP-1 expression in osteocyte-like cell, while not be detected in MC3T3-E1 (S1 Fig). With the cutoff criteria of a fold-change >1.5 and P < 0.05, we identified 724 differentially expressed mRNAs and circRNAs between control and N.g MDP–treated samples, with 579 up-regulated and 145 down-regulated genes in differentially expressed mRNAs and 309 up-regulated and 415 down-regulated circRNAs (Fig 1). The top-10 dysregulated mRNAs (Table 1) and circRNAs (Table 2) were summarized based on P values. Bioinformatics analysis of the interactions (Table 3) and gene pathways between the two samples have been submitted. STRING database analysis identified 717 nodes and 1218 edges (S2 Fig).

**Table 1:** Top 10 dysregulated mRNAs in MLO-Y4 cells treated with N.g MDP.

Gene	Fold-change	Status	P	FDR
S100a10	0.648097	Down	2.15E-76	4.43E-74
Rpl39l	0.657591	Down	7.93E-65	1.42E-62
Eif4a2	1.567235	Up	1.11E-53	1.77E-51
Rps27l	0.644698	Down	1.62E-52	2.55E-50
Cox7c	0.606489	Down	1.91E-51	2.98E-49
Atp5e	0.607108	Down	1.57E-45	2.32E-43
Uqcrh	0.619202	Down	1.12E-44	1.60E-42
Luc7l2	1.675182	Up	1.01E-35	1.20E-33
Nfat5	1.643251	Up	5.42E-34	6.03E-32
Cox6c	0.628503	Down	7.00E-32	7.49E-30

**Table 2:** Top 10 dysregulated circRNAs in MLO-Y4 cells treated with N.g MDP.

circRNA_ID	circBase_ID	geneID	Status	P
chr18:83124916|83126841	mmu_circ_0000909	Zfp516	Down	4.87E-89
chr11:108873006|108873262		U6,SNORA70,SCARNA4,7SK,snoR38,U3	Down	1.13E-85
chr16:11144101|11144327		U6,7SK,SNORA17,5S_rRNA,U1,Zc3h7a	Down	1.49E-84
chr17:39985361|39985627		AY036118	Down	5.71E-70
chr5:137753102|137753461		Trip6	Down	4.78E-69
chr7:17468476|17468859	mmu_circ_0001545	Dact3	Down	3.31E-68
chr11:104285236|104286607	mmu_circ_0000331	1700081L11Rik	Down	7.54E-63
chr19:4784724|4788042	mmu_circ_0000918	Rbm14	Down	9.89E-63
chr3:51111972|51129957	mmu_circ_0001125	Elf2	Up	3.83E-59
chr16:15887401|15887572		Cebpd	Down	1.24E-57

**Table 3:** The significantly enriched pathways associated with 724 mRNAs in the N.g MDP–treated osteocyte model.

Category	Pathway	Description	Gene count	P
Biological process	Regulation of transcription, DNA-templated	GO:0006355	127	2.27E-09
	Ureteric bud development	GO:0001657	7	0.004772
	Transcription, DNA-templated	GO:0006351	82	0.007932
	Necroptotic process	GO:0070266	4	0.007997
	Inhibitory postsynaptic potential	GO:0060080	4	0.007997
Cellular component	Intracellular	GO:0005622	94	2.07E-08
	Ciliary transition zone	GO:0035869	7	2.31E-04
	Nucleus	GO:0005634	224	0.008896
	Mitochondrial proton-transporting ATP synthase complex	GO:0005753	4	0.019214
	Endoplasmic reticulum	GO:0005783	56	0.033769
Molecular function	Nucleic acid binding	GO:0003676	95	3.96E-14
	Metal ion binding	GO:0046872	182	3.83E-12
	Transcription factor activity, sequence-specific DNA binding	GO:0003700	50	3.88E-04
	DNA binding	GO:0003677	89	4.32E-04
	Syntaxin binding	GO:0019905	8	0.017731

### Construction of the circRNA–miRNA–mRNA triple network

**Fig 2:**
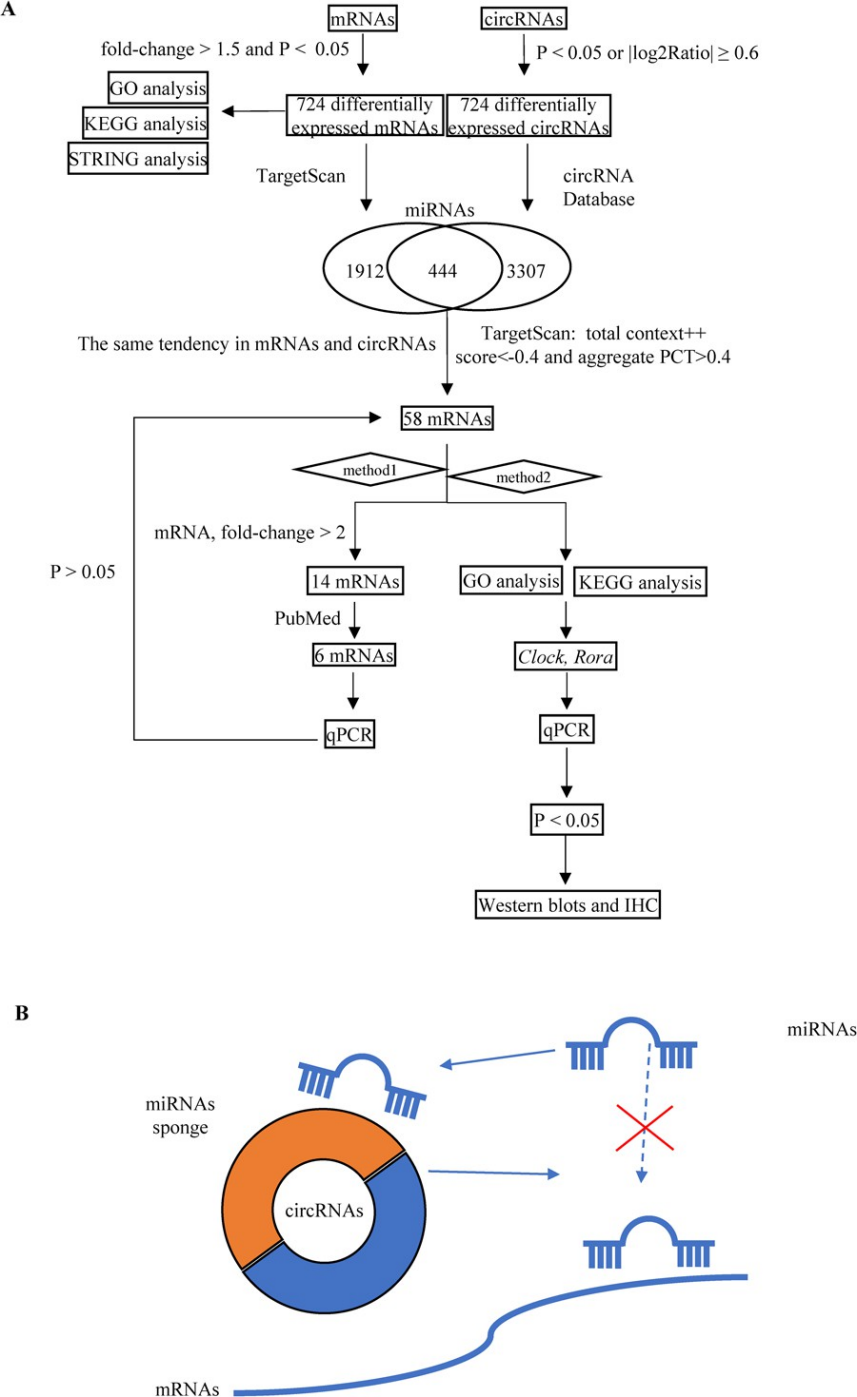
Combination of mRNA and circRNA databases to construct the circRNA–miRNA–mRNA triple network. (A) Analysis method for constructing circRNA-miRNA-mRNA networks. (B) Diagram of the triple networks.

**Fig 3:**
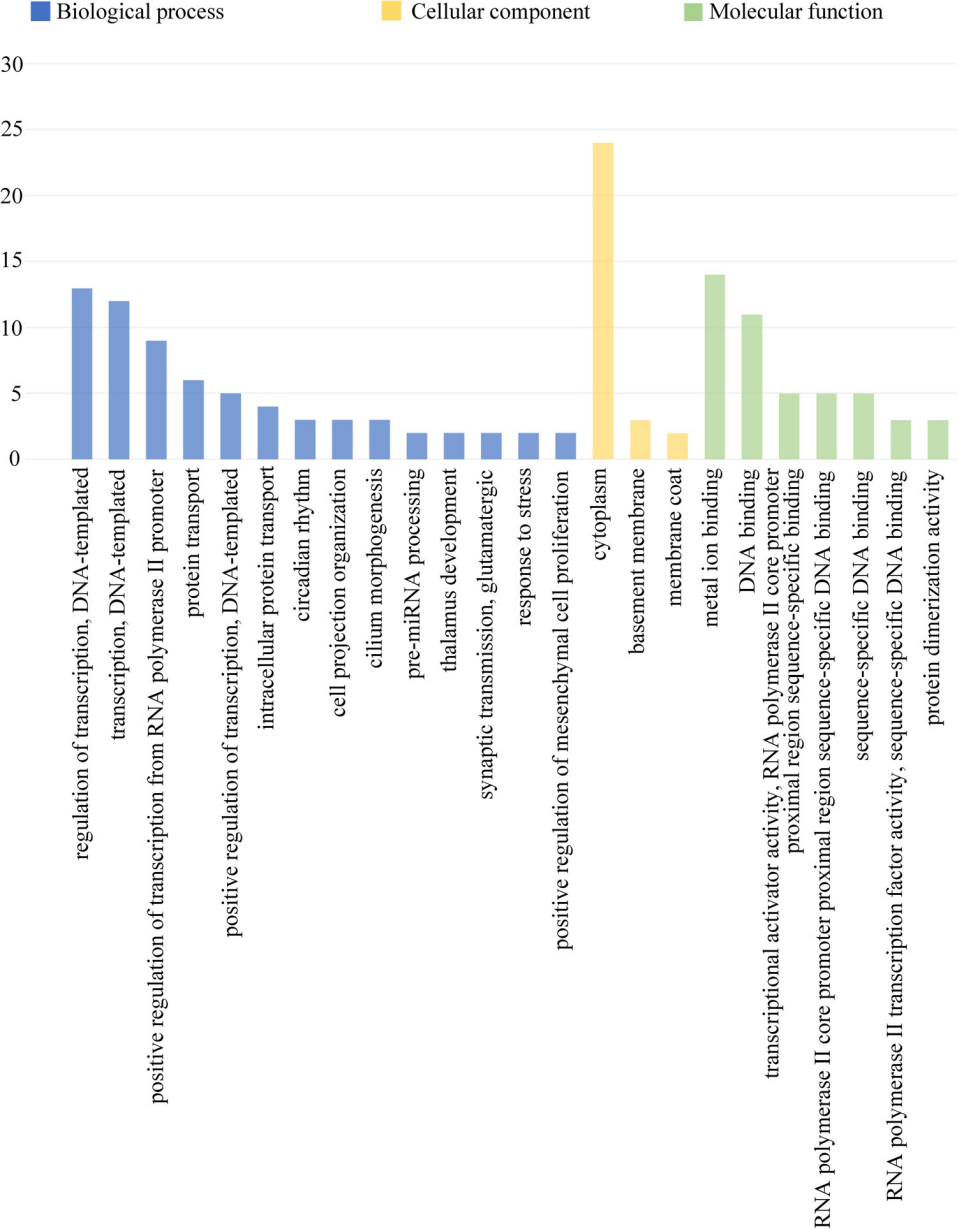
Bioinformatics analysis of the interactions among the differentially expressed genes in the N.g MDP–treated osteocytes. The most significantly enriched GO (−log10 (P value)) terms of mRNA gene symbols according to biological process are marked in blue; cellular component in yellow; and molecular function in green. GO, gene ontology.

**Fig 4:**
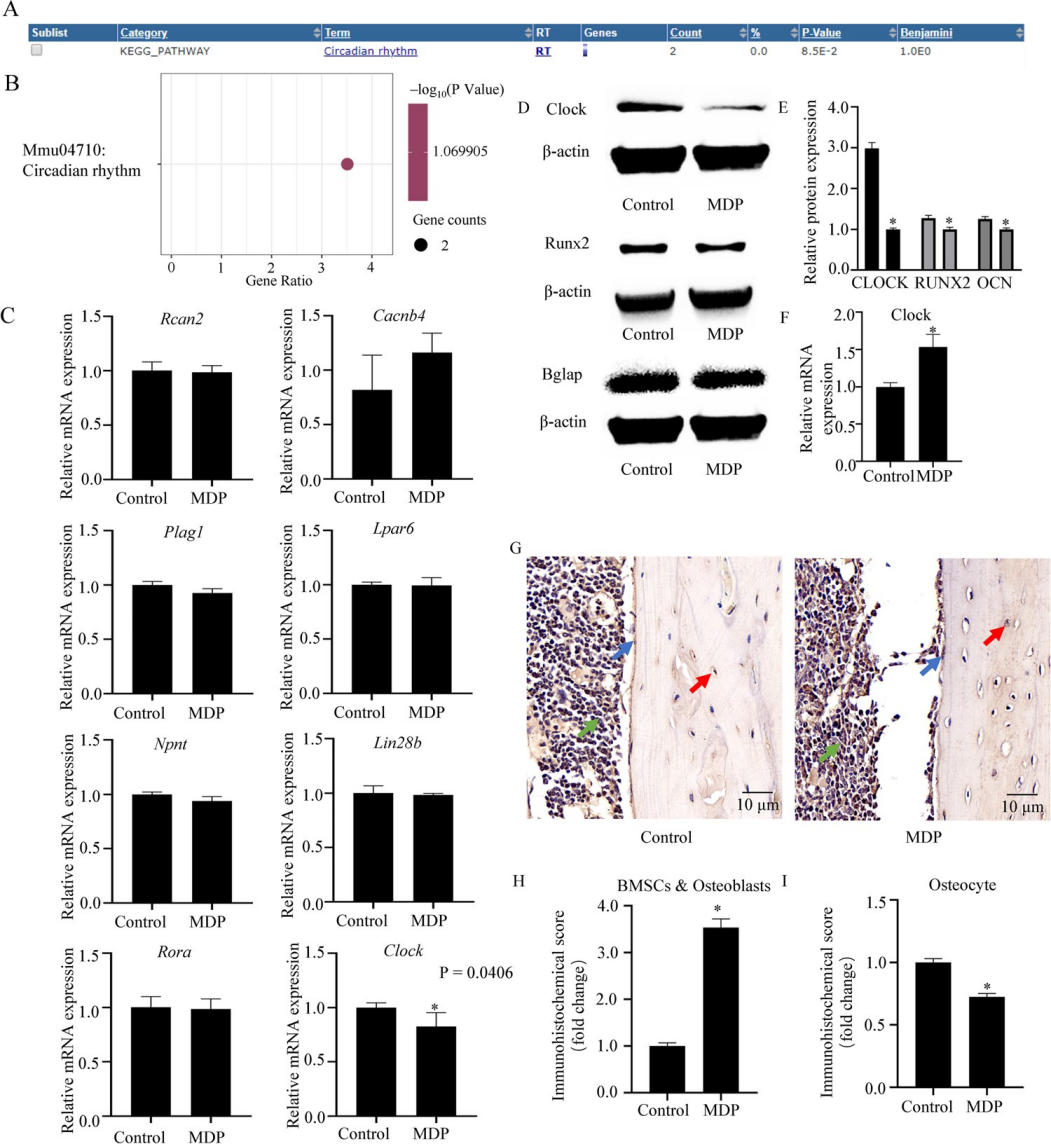
Bioinformatics analysis of the gene pathways that were enriched in the N.g MDP–treated osteocytes and verification *in vitro*. (A and B) Pathway enrichment analysis showed that the differentially expressed genes, including clock and Rora, were associated with Circadian rhythm pathways. The size of the nodes and the intensity of the color indicate the gene number and mean P value. (C) qRT-PCR showed decreased expression of clock mRNA, n = 3. (D) Western blots showed decreased expression of CLOCK, RUNX2, and BGLAP. (E) Quantitative analysis of the expression of CLOCK protein. n = 3. (F) qRT-PCR showed increased expression of *clock* mRNA after osteogenic induction. (G) In vivo, immunohistochemistry showed increased CLOCK expression in bone mesenchymal stem cells (BMSCs) and osteoblasts, but decreased expression in osteocytes. Scale bar = 10 μm. (H and I) Quantitative analysis of immunohistochemical score of (G). Green arrow, BMSCs; blue arrow, osteoblast; red arrow, osteocyte; Control, the sample was treated with culture medium in vitro or saline only in vivo; MDP, the sample was treated with 1 μg/ml N.g MDP for 36 h in vitro or 2 μg N.g MDP dissolved in 100 μl of saline solution for 10 days in vivo.

To investigate ceRNA regulation in N.g MDP–treated osteocyte-like cell and characterize the osteoblastic-related circRNA–miRNA–mRNA triple network, we used TargetScan (http://www.targetscan.org/) databases to predict miRNA–mRNA and obtained 1912 miRNA–mRNA interaction pairs. Using the circRNA database provided by Guangzhou Forevergen Biosciences Co., Ltd., we obtained 3307 circRNA–miRNA interaction pairs. Furthermore, we screened out the expression of mRNA and circRNA, which were consistent. Using the cutoff criteria of total context++ score < -0.4 and aggregate probability of conserved targeting >0.4 in the TargetScan software, we obtained 58 circRNA–miRNA–mRNA interaction pairs (Fig 2). We first selected mRNAs with expression meeting a fold-change threshold >2 and identified 14 differentially expressed genes (*Dbndd1*, *Rcan2*, *Kif24*, *Cacnb4*, *Atf7ip2*, *Lin28b*, *5031414D18Rik*, *Npnt*, *Lpar6*, *Mybl1*, *Ttc14*, *Zc3h12b*, *Plag1*, and *Alg6*). In related searches of PubMed, we found six that were osteoblastic-related (*Rcan2*, *Cacnb4*, *Plag1*, *Lpar6*, *Npnt*, and *Lin28b*). We also assessed a selection of 58 mRNAs by GO (Table 4) and KEGG analysis (Figs 3 and 4A and 4B). Bioinformatics analysis of gene pathways identified “Circadian rhythm” as uniquely associated with the differentially expressed mRNA, including *Clock* and *Rora*.

**Table 4:** The significantly enriched pathways associated with 58 mRNAs in the N.g MDP–treated osteocyte model.

Category	Pathway	Description	Gene count	P
Biological process	Positive regulation of transcription from RNA polymerase II promoter	GO:0045944	9	0.004934
	Transcription, DNA-templated	GO:0006351	12	0.010709
	Regulation of transcription, DNA-templated	GO:0006355	13	0.016459
	Protein transport	GO:0015031	6	0.021561
	Intracellular protein transport	GO:0006886	4	0.024918
Cellular component	Basement membrane	GO:0005604	3	0.027222
	Cytoplasm	GO:0005737	24	0.074427
	Membrane coat	GO:0030117	2	0.076426
Molecular function	Transcriptional activator activity, RNA polymerase II core promoter proximal region sequence-specific binding	GO:0001077	5	0.00508
	RNA polymerase II core promoter proximal region sequence-specific DNA binding	GO:0000978	5	0.01353
	DNA binding	GO:0003677	11	0.017334
	RNA polymerase II transcription factor activity, sequence-specific DNA binding	GO:0000981	3	0.074094
	Metal ion binding	GO:0046872	14	0.077303

### De-regulation of *Clock* expression in N.g MDP–treated osteocyte-like cell *in vitro* and in femurs of N.g MDP–treated mice *in vivo*

To verify *in vitro* and *in vivo* the accuracy of the filtered results for the differentially expressed mRNAs, osteocyte-like cell was treated with or without (control) 1 μg/ml N.g MDP for 36 h, and quantitative reverse-transcription polymerase chain reaction (qRT-PCR) was used to measure and compare the expression of the identified genes in control and treated samples. We found that the expression of *Clock* mRNA was decreased approximately 20% (P < 0.05), but found no other significant differences between the two samples (Fig 4C).

Western blots showed an obviously decreasing amount of CLOCK protein (P < 0.05) (Fig 4D and 4E), along with a tendency for expression of the osteogenic differentiation biomarkers *Runx2* and *Bglap* to decline. The qRT-PCR showed that *Clock* mRNA was upregulated in BMSCs under osteogenic induction (Fig 4F). To illustrate the expression of CLOCK protein *in vivo*, mice were infused with 2 μg N.g MDP dissolved in 100 μl of saline solution once a day for 10 days, and the control group was infused with 100 μl saline only. Immunohistochemistry showed increased CLOCK protein expression in BMSCs and osteoblasts from femurs of N.g MDP–treated animals, but decreased expression in osteocytes (Fig 4G–4I).

## Discussion

In this study, we analyzed RNA-seq results of mRNA and circRNA, and integrated these findings with bioinformatics analysis to construct a circRNA–miRNA–mRNA triple network. According to the two different analytical methods we applied, N.g MDP may control the process of osteoblastic differentiation through regulation of *Clock* expression in osteocyte-like cell, suggesting a potential therapeutic target in leprosy-related bone loss.

In the context of bone metabolism, RNA-seq has influenced every aspect of understanding of genomic function [34,35]. Using a combination of large-scale genome and transcriptome studies with bioinformatic analysis, Xu and colleagues found that targeting the SLIT3 pathway could represent a new approach to treating bone loss [36]. However, no related study has demonstrated an interaction between ncRNA and leprosy. As noted, *M*. *leprae* cannot be cultured *in vitro*, representing an obstacle to researching bone remodeling mechanisms in this disease [16,37,38].

MDP is a component of the cell wall and the minimal essential structure for eliciting immunological effects [16]. To further investigate the relationship between MDP and ncRNA, we developed an *in vitro* MDP-treated osteocyte-like cell model and identified several differentially expressed mRNAs and circRNAs. However, because we could not verify all of these differentially expressed genes in experimental analysis, we used TargetScan and the circRNA database (provided by Guangzhou Forevergen Biosciences Co., Ltd.) to predict the target miRNA and construct a circRNA–miRNA–mRNA triple network. The circRNA–miRNA–mRNA triple network was wildly applied in research, Chen X et al. found that CircRNA_28313/miR-195a/CSF1 could regulate osteoclastic differentiation in OVX–induced bone absorption in mice [39], and Shen WX et al. revealed that CircFOXP1/miR-33a-5p/FOXP1 could promote osteogenic differentiation and CircFOXP1 can be used as a potential osteoporosis therapeutic target [40]. Based on the predicted results, we screened out the differentially expressed genes using a fold-change cutoff of >2.0 for mRNA [41]. We narrowed the list to six differentially expressed mRNAs using a PubMed search and to two differentially expressed mRNAs using GO and KEGG analysis. We confirmed decreased expression of the *Clock* gene, one of the two identified with the latter approach, but that expression of *Rcan2*, *Cacnb4*, *Plag1*, *Lpar6*, *Npnt*, *Lin28b*, and *Rora* did not differ between treated and untreated samples (Fig 4C). Thus, this circRNA–miRNA–mRNA triple network could provide a new analytical approach to elucidating the relationship between N.g MDP and bone remodeling.

The circadian clock is a key factor in the circadian system, which is associated with cell function, metabolic state, and life expectancy [42,43]. Bone remodeling could be subject to circadian regulation [44]. Previously research has indicated that mice lacking Clock or brain and muscle Arnt -like protein 1 (Bmal1) have a reduced bone mass [45]. A recent study demonstrated that the circadian clock could regulate bone formation through transcriptional control of the 1,25-dihydroxy-vitamin D3 receptor PDIA3 [46]. Decreasing of the bone mineral density and increasing risk of patients developing osteoporosis would happen, if the circadian rhythm has disordered [47]. Bglap and Runx2 also displayed rhythmicity with the expression of Clock gene [48]. BMAL1 could influence numbers of key factors in skeletal development, such as Runx2/Osterix and HIF1α-VEGF signaling pathway [49]. Our results showed decreased expression of *Clock*, along with expression of the osteogenic biomarkers Runx2 and Bglap (Fig 4D), while the expression of *Clock* was increased in BMSCs under osteogenic induction. Yuan HP et al. found that intraperitoneal injection of MDP in ApoE^−/−^ mice could reduce alveolar bone loss which expose in *Porphyromonas gingivalis* [50]. Park OJ et al. revealed that MDP could induce bone formation and osteoblast activation by upregulating the expression of Runx2 and ALP in BMSCs and preosteoblasts, and micro-CT showed increased trabecular bone in the femur of MDP-administered mice [51]. However, the regulatory effect of MDP on osteocyte still not be reported. Immunohistochemical analysis indicated that expression of the CLOCK protein was significantly increased in murine femur BMSCs and osteoblasts, while decreased in osteocytes in exposed mice (Fig 4E). The paradoxical results are possibly because of negative feedback regulation of osteocytes to osteoblasts and BMSCs [52], and indicate that different cells induce different responses *in vivo* and *in vitro*. Previous study has generated *Irs-1*-deficient *Irs*-*1*^smla/smla^ mice, and in BMSCs derived from *Irs-1*-null mice, the expression of COL1A2 was decreased, then, osteocytes promote osteogenesis through negative feedback regulation [53]. However, the candidate miRNA and circRNA involving in our RNA-seq, miR-322-5p and mmu_circ_0006240, has not been reported. In general, these results suggest a crucial role for Clock in bone remodeling.

In summary, based on the results of transcriptome studies and bioinformatic analysis, we have characterized a circRNA–miRNA–mRNA triple network and screened out the key factors in the N.g MDP–treated osteocyte-like cell. Our approach potentially represents a new analytical method for elucidating the mechanism of bone remodeling in leprosy, and the circRNA-miRNA-mRNA could filter the important factors that might change the presentation of the diseases.

## Supporting information

S1 Fig MLO-Y4 cultured and examined. (A) MLO-Y4 under a light microscope. (B) Western blots showed MLO-Y4 could express DMP-1 protein.
(TIF)

S2 Fig Bioinformatics analysis between control and treated samples. (A) Bioinformatics analysis of the interactions between the differentially expressed genes in the N.g MDP–treated osteocytes. (B) Bioinformatics analysis of the gene pathways that were enriched in the N.g MDP–treated osteocytes. (C) Protein–protein interaction (PPI) analysis in the N.g MDP–treated osteocytes.
(TIF)
