# Supplementary figures and images for: A comprehensive analysis of the circRNA–miRNA–mRNA network in osteocyte-like cell associated with Mycobacterium leprae infection
Source: PLoS Negl Trop Dis. 2022 May 2;16(5):e0010379. doi: 10.1371/journal.pntd.0010379 (PMC9098081; doi:10.1371/journal.pntd.0010379)

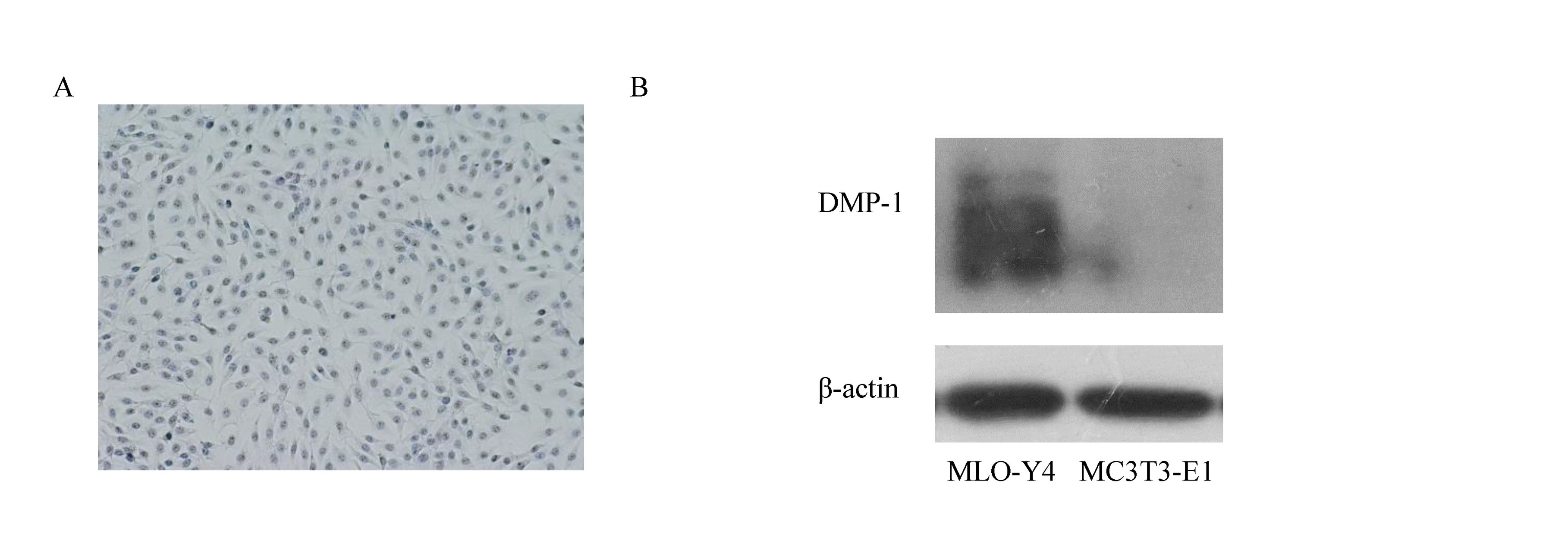

Supplement: S1 Fig — (A) MLO-Y4 under a light microscope. (B) Western blots showed MLO-Y4 could express DMP-1 protein. (TIF) [file pntd.0010379.s001.tif]

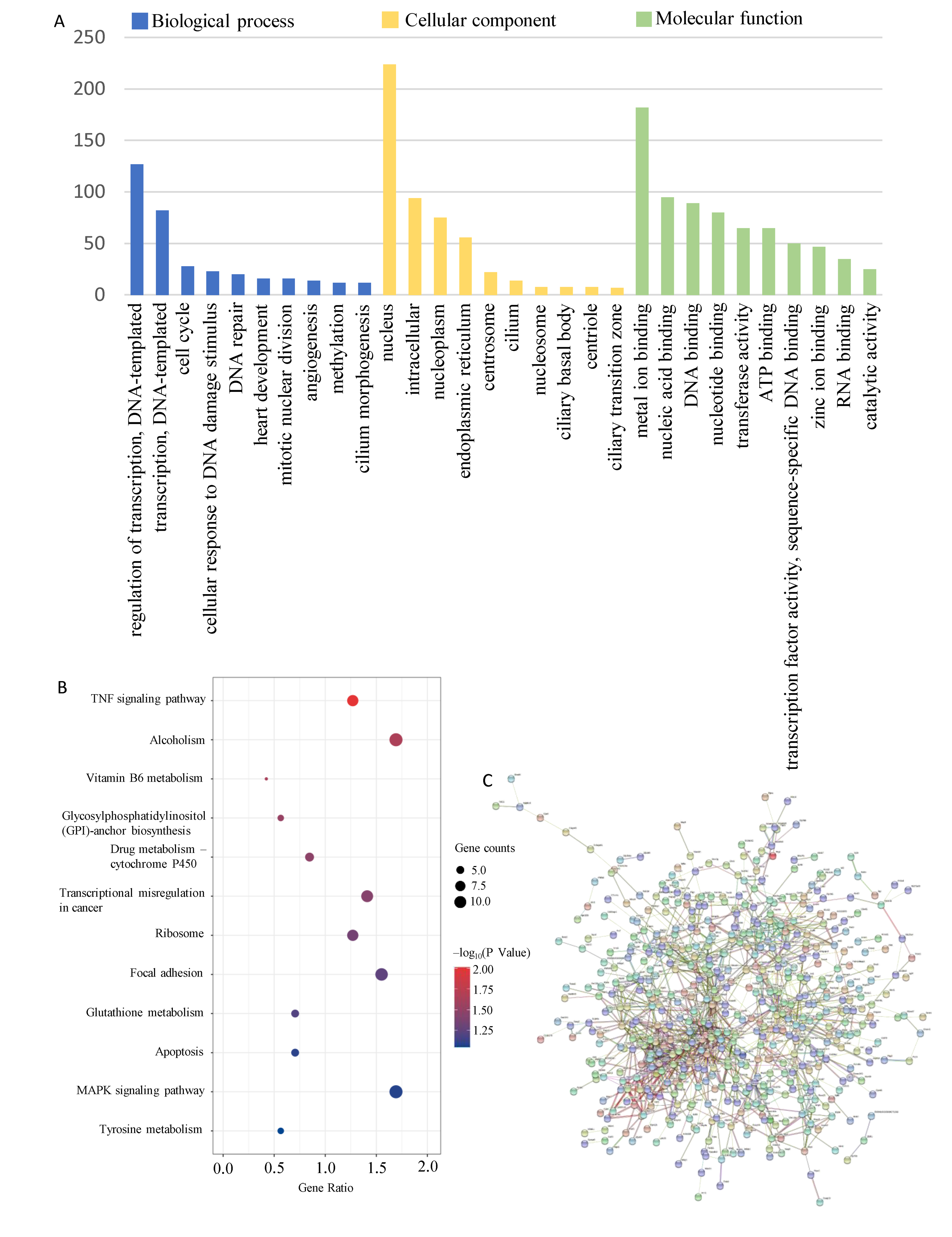

Supplement: S2 Fig — (A) Bioinformatics analysis of the interactions between the differentially expressed genes in the N.g MDP–treated osteocytes. (B) Bioinformatics analysis of the gene pathways that were enriched in the N.g MDP–treated osteocytes. (C) Protein–protein interaction (PPI) analysis in the N.g MDP–treated osteocytes. (TIF) [file pntd.0010379.s002.tif]
